# New biomarker in the early diagnosis of breast cancer: adiponectin, asprosin, adropin, and resistin

**DOI:** 10.1186/s12885-026-16183-z

**Published:** 2026-05-22

**Authors:** Ömer Çelik, Halit Özgül, Sibel Kulaksızoğlu, Osman Zekai Öner, Barış Rafet Karakaş, Remzi Can Çakır, Zinet Asuman Arslan Onuk, Uğur Bilge

**Affiliations:** 1 Department of General Surgery, Kastamonu Training and Research Hospital, Kastamonu, Turkey; 2 Department of General Surgery, Memorial Antalya Hospital, Antalya, Turkey; 3 Department of Biochemistry, Antalya Training and Research Hospital, University of Health Sciences, Antalya, Turkey; 4 Department of General Surgery, Antalya Training and Research Hospital, University of Health Sciences, Antalya, Turkey; 5 Department of General Surgery, Bursa City Hospital, University of Health Sciences, Bursa, Turkey; 6 Department of Anesthesiology and Reanimation, Antalya Training and Research Hospital, University of Health Sciences, Antalya, Turkey; 7 Department of Biostatistics and Medical Informatics, Faculty of Medicine, Akdeniz University, Antalya, Turkey

**Keywords:** Breast cancer, Early diagnosis, Adiponectin, Adropin, Asprosin, Resistin

## Abstract

**Background:**

Early diagnosis of breast cancer remains difficult despite advances in imaging because false-positive and false-negative findings still occur. Circulating biomarkers reflecting metabolic and inflammatory changes may provide complementary information. Adiponectin, adropin, asprosin, and resistin have been suggested as candidates, yet their comparative diagnostic performance in breast cancer has not been adequately defined.

**Methods:**

This study included ninety women aged 18–60 years allocated to malignant breast cancer, benign breast disease, and healthy control groups (*n* = 30 each). All malignant cases were invasive ductal carcinoma. Serum adiponectin, adropin, asprosin, and resistin concentrations were measured using enzyme-linked immunosorbent assay. Group comparisons used parametric or non-parametric tests as appropriate. Diagnostic accuracy was evaluated by receiver operating characteristic analysis, optimal cut-off values by the Youden index. When appropriate, post hoc pairwise comparisons were performed following overall group comparisons to identify between-group differences.

**Results:**

Adiponectin levels were significantly lower, whereas adropin, asprosin, and resistin levels were significantly higher in malignant cases than in benign disease and controls (all *p* < 0.001). Receiver operating characteristic analysis showed good to excellent discrimination for adropin (AUC 0.893), asprosin (AUC 0.906), and resistin (AUC 0.908), while adiponectin demonstrated moderate accuracy (AUC 0.786). Adropin, asprosin, and resistin correlated strongly with each other and showed moderate relationships with tumor size and stage; adiponectin displayed weaker correlations.

**Conclusion:**

Adropin, asprosin, and resistin show promising diagnostic value for early breast cancer and outperform adiponectin. These biomarkers may complement imaging in clinical evaluation, although larger prospective studies are needed to confirm clinical applicability and to establish standardized thresholds for routine clinical practice across diverse patient populations.

## Introduction

Breast cancer is the leading cause of cancer-related mortality among women worldwide [1]. In 2020, breast cancer was the most diagnosed cancer among women, with 2.3 million reported cases and 685,000 deaths globally [2]. Radiological screening methods are widely used for breast cancer screening worldwide, including mammography, ultrasonography, and magnetic resonance imaging (MRI) for early diagnosis [3]. Mammography, the most used screening method, has certain limitations, such as false-positive and false-negative findings, which may lead to diagnostic errors [4]. Therefore, blood-based molecular biomarkers with high diagnostic performance may serve as an alternative to enhance and complement the currently used imaging techniques for breast cancer detection.

Adiponectin is an adipocytokine secreted by adipose tissue that plays a role in various physiological processes, including insulin sensitivity, anti-inflammatory effects, and energy metabolism [5]. Research suggests that adiponectin levels may be associated with the development and progression of breast cancer. Several studies have demonstrated a correlation between low serum adiponectin levels and an increased risk of breast cancer. Notably, studies conducted in postmenopausal women have found that serum adiponectin levels in breast cancer patients are significantly lower compared to control groups [6].

Asprosin is an adipokine secreted by white adipose tissue that promotes glucose production in the liver during fasting [7]. Additionally, it has appetite-stimulating effects. Existing literature suggests that asprosin levels may be associated with various metabolic diseases and certain types of cancer [8, 9]. However, specific studies directly investigating the relationship between asprosin and breast cancer remain limited.

Adropin is a peptide hormone involved in the regulation of energy homeostasis, lipid and glucose metabolism, and cardiovascular functions [10]. Studies on cancer suggest that adropin levels may vary in certain cancer types. In patients with endometrial cancer, lower adropin levels have been observed, and it has been proposed that this may play a role in the pathogenesis of the disease [11].

Resistin is a pro-inflammatory cytokine first identified in 2001, primarily secreted by macrophages in humans. It plays a significant role in processes such as cancer cell proliferation, metastasis, angiogenesis, and inflammation. Elevated serum resistin levels have been associated with disease progression and poor prognosis in various types of cancer. Although studies investigating the role of resistin in breast cancer are limited, some research suggests that resistin levels may be linked to breast cancer development [12].

While various studies in the literature have explored the potential of adiponectin, asprosin, adropin, and resistin as predictive biomarkers for breast cancer, conclusive results have yet to be obtained. Therefore, in our study, we aimed to investigate and compare the effects of adiponectin, asprosin, adropin, and resistin in the early diagnosis of breast cancer.

## Methods

### Study design

This study was designed to investigate the diagnostic value of novel biomarkers in the early diagnosis of breast cancer. A total of 90 female patients aged between 18 and 60 years were included in the study.

Patients were categorized into three groups based on their clinical and histopathological diagnoses:

The study population consisted of three groups. Healthy group (*n* = 30) included women with no clinical or radiological evidence of breast pathology, confirmed by normal breast ultrasound and/or mammography performed within the past six months. Benign group (*n* = 30) comprised women with breast lesions categorized as BIRADS 2, 3, or 4, with lesions definitively diagnosed as benign through radiological and/or histopathological evaluation (e.g., fibroadenoma or fibrocystic changes). Malignant group (*n* = 30) included patients with newly diagnosed invasive breast cancer confirmed by core needle biopsy or surgical pathology. All malignant cases were classified as invasive ductal carcinoma. Although histological subtypes and tumor stages were recorded, subgroup analysis was not performed due to sample size limitations. Hormone receptor status was available for 60% of the malignant cases.

Exclusion criteria included the presence of an additional malignancy, distant metastasis, receipt of neoadjuvant chemotherapy, and diagnosis of a hereditary cancer syndrome. Patients with known active metabolic or inflammatory disorders (including diabetes mellitus, metabolic syndrome, and chronic inflammatory diseases) were excluded in order to minimize potential confounding effects on circulating adipokine levels.

### Biochemical analysis

Five milliliters of venous blood were collected into tubes containing spray-dried clot activator and gel separator. The samples were centrifuged at 4000 rpm for 10 min using a Nüve NF800 centrifuge. Following centrifugation, the serum was transferred into Eppendorf tubes and stored at − 80 °C until the day of analysis. All assays were performed using the solid-phase enzyme-linked immunosorbent assay (ELISA) method. Serum levels of resistin, adropin, asprosin, and adiponectin were measured using commercially available ELISA kits (SunRed Bio, Shanghai, China), including the Human Resistin ELISA Kit (Cat. No: 201-12-0339), Human Adropin ELISA Kit (Cat. No: 201-12-3107), Human Asprosin ELISA Kit (Cat. No: 201-12-7193), and Human Adiponectin ELISA Kit (Cat. No: 201-12-1551). The intra-assay coefficient of variation (CV) for each test was less than 10%, indicating high reproducibility.

### Statistical analysis

Statistical analyses were performed using IBM SPSS Statistics for Windows, version 23.0 (IBM Corp., Armonk, NY, USA). The normality of continuous variables was assessed using both visual methods (histograms and probability plots) and analytical methods (Shapiro–Wilk test). Descriptive statistics were presented as numbers and percentages for categorical variables, as mean ± standard deviation (SD) for normally distributed continuous variables, and as median (min–max) for non-normally distributed continuous variables. Comparisons of continuous variables among the three groups were performed using one-way analysis of variance (ANOVA) for normally distributed variables and the Kruskal–Wallis test for non-normally distributed variables. When a statistically significant overall difference was detected with the Kruskal–Wallis test, post hoc pairwise comparisons were conducted using the Mann–Whitney U test with Bonferroni correction, and the adjusted significance level was set at *p* < 0.017 (0.05/3). Post hoc pairwise comparisons after ANOVA were planned only if the overall ANOVA result was statistically significant; however, no such comparison was required because the only ANOVA-tested variable, age, was not significant.

To evaluate the diagnostic performance of adiponectin, adropin, asprosin, and resistin for identifying malignant breast cancer, receiver operating characteristic (ROC) curve analysis was performed. For ROC analyses, patients with benign breast disease and healthy controls were combined into a single non-malignant group. The diagnostic performance of each variable was assessed using the area under the ROC curve (AUC). AUC values between 0.9 and 1.0 were considered excellent, 0.8–0.9 good, 0.7–0.8 fair, 0.6–0.7 poor, and 0.5–0.6 failed [13]. Optimal cut-off values were determined using the Youden Index, calculated as max (sensitivity + specificity – 1) [14, 15]. Following the ROC analysis results, the AUC and cut-off values, sensitivity and specificity of these cut-offs, likelihood ratio (LHR), positive predictive value (PPV) and negative predictive value (NPV) were calculated.

Correlations between serum biomarker levels were assessed using Spearman’s rank correlation analysis. Correlation strength was interpreted according to commonly accepted criteria (weak: <0.40, moderate: 0.40–0.69, strong: ≥0.70) [16]. Multivariate logistic regression analysis was performed to evaluate the independent effects of the biomarkers on malignancy. Due to high correlations among adropin, asprosin, and resistin, these variables were not included in the same model to avoid multicollinearity. Instead, three separate models were constructed, each including adiponectin in combination with one of the other biomarkers (adropin, asprosin, or resistin). A p-value < 0.05 was considered statistically significant.

## Results

A total of 90 participants were included in the study, comprising patients with malignant breast cancer (*n* = 30), benign breast disease (*n* = 30), and healthy controls (*n* = 30). All patients included in the study were female. All malignant breast tumors were of ductal histology. Tumor laterality was evenly distributed between the right and left breasts. In the malignant breast cancer group, 15 tumors (50%) were located in the right breast and 15 tumors (50%) in the left breast. The benign breast disease group showed the same distribution, with 15 right-sided (50%) and 15 left-sided (50%) lesions. There was no statistically significant difference in age among the three groups (49.3 ± 7.9, 47.9 ± 8.2, and 45.3 ± 7.7 years for malignant, benign, and healthy groups, respectively; *p* = 0.151). Similarly, BMI values did not differ significantly between groups (*p* = 0.160. Serum adiponectin levels differed significantly among the three groups (*Kruskal–Wallis test*, *p* < 0.001). Post hoc analysis with Bonferroni-adjusted Mann–Whitney U tests demonstrated that adiponectin levels were significantly lower in both malignant breast cancer and benign breast disease groups compared with healthy controls (both *p* < 0.001), whereas no significant difference was observed between malignant and benign groups (*p* = 0.110). A significant overall difference was also observed for adropin levels among the study groups (*p* < 0.001). Post hoc comparisons revealed that adropin levels were significantly higher in the malignant breast cancer group compared with both benign breast disease and healthy control groups (both *p* < 0.001). In addition, adropin levels were significantly higher in the benign group compared with healthy controls (*p* = 0.006). Asprosin levels showed a significant difference across the three groups (*p* < 0.001). Post hoc analysis indicated that asprosin levels were significantly elevated in the malignant breast cancer group compared with both benign breast disease and healthy controls (both *p* < 0.001). Moreover, patients with benign breast disease also exhibited significantly higher asprosin levels than healthy controls (*p* = 0.001). Similarly, resistin levels differed significantly among the groups (*p* < 0.001). Post hoc comparisons demonstrated significantly higher resistin levels in the malignant breast cancer group compared with both benign breast disease and healthy controls (both *p* < 0.001). However, no statistically significant difference in resistin levels was observed between the benign breast disease and healthy control groups (*p* = 0.172) (Table 1).

**Table 1:** Comparison of demographic characteristics and serum levels of adiponectin, adropin, asprosin, and resistin among malignant breast cancer, benign breast disease, and healthy control groups

Variables	Malignant breast cancer, *n* = 30	Benign, *n* = 30	Healthy, *n* = 30	*p*
Age, years Mean ± SD	49.3 ± 7.9	47.9 ± 8.2	45.3 ± 7.7	0.151*
BMI, kg/m² Median (min-max)	26.5 (20.8–39.0)	27.8 (22.9–34.8)	25.6 (21.3–30.4)	0.160**
Adiponectin, µg/mL Median (min-max)	120.5 (5.0-247.0)	142.0 (74.0-564.0)	476.0 (139.0-1157.0)	< 0.001** ^***a***^
Adropin, ng/mL Median (min-max)	18.0 (4.0–55.0)	5.0 (4.0–28.0)	4.0 (1.0–9.0)	< 0.001** ^***b***^
Asprosin, ng/mL Median (min-max)	239.5 (41.0-827.0)	44.0 (26.0-407.0)	33.5 (5.0-100.0)	< 0.001** ^***c***^
Resistin, ng/mL Median (min-max)	43.5 (12.0–63.0)	12.0 (7.0–51.0)	10.5 (1.0–23.0)	< 0.001** ^***d***^

*SD* Standard deviation

Data in bold is statistically significant

* One-way ANOVA

** Kruskal–Wallis test: Post hoc pairwise comparisons were performed using the Mann–Whitney U test with Bonferroni correction for biomarkers showing a significant overall difference in the Kruskal–Wallis test. The adjusted significance level was set at*p* < 0.017 (0.05/3. Superscripts (a–d) indicate statistically significant pairwise comparisons between groups according to the Bonferroni-adjusted Mann–Whitney U test.)

^a^ Post hoc pairwise comparisons: Malignant breast cancer vs. Benign, *p* = 0.110; Malignant breast cancer vs. Healthy, *p* < 0.001; Benign vs. Healthy, *p* < 0.001

^b^ Post hoc pairwise comparisons: Malignant breast cancer vs. Benign, p < 0.001; Malignant breast cancer vs. Healthy, *p* < 0.001; Benign vs. Healthy, *p* = 0.006

^c^ Post hoc pairwise comparisons: Malignant breast cancer vs. Benign, p < 0.001; Malignant breast cancer vs. Healthy, *p* < 0.001; Benign vs. Healthy, *p* = 0.001

^d^ Post hoc pairwise comparisons: Malignant breast cancer vs. Benign, *p* < 0.001; Malignant breast cancer vs. Healthy, *p* < 0.001; Benign vs. Healthy,*p* = 0.172

**Fig. 1:**
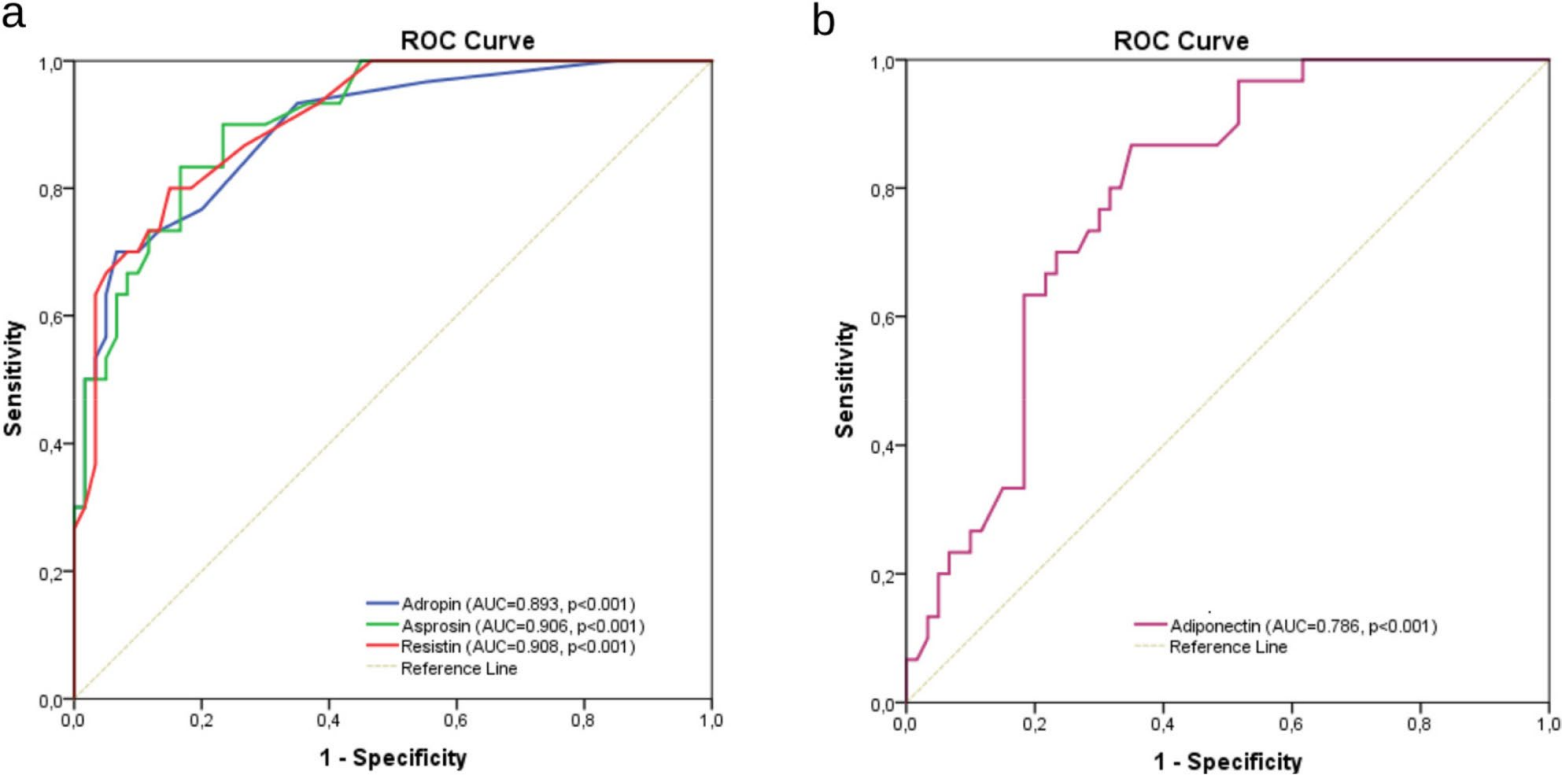
ROC Curve Analysis of Adiponectin, Adropin, Asprosin, and Resistin for the Identification of Malignant Breast Cancer (**a**: Higher values indicate a more diagnostically positive result for identifying malignancy. **b**: Lower values indicate a more diagnostically positive result for identifying malignancy.)

The clinicopathological characteristics of malignant breast tumors are summarized in Table 2. For ROC curve analyses, benign breast disease and healthy controls were combined into a single non-malignant group and compared with patients with malignant breast cancer. Optimal cut-off values for each biomarker were determined using the Youden index, and diagnostic performance was further evaluated using positive likelihood ratios (+ LHR) to assess clinical discriminative strength. This approach was adopted to evaluate the ability of the investigated biomarkers to discriminate malignant lesions from non-malignant conditions in a clinically relevant binary setting. ROC analyses demonstrated good to excellent diagnostic performance for adropin, asprosin, and resistin, with AUC values of 0.893, 0.906, and 0.908, respectively (all *p* < 0.001) (Fig. 1a), while adiponectin showed moderate discriminative ability (AUC = 0.786, *p* < 0.001) (Fig. 1b). The optimal cut-off values for distinguishing malignant from non-malignant cases were ≤ 140.0 for adiponectin (sensitivity 70.0%, specificity 76.7%, PPV 60.0%, NPV 83.6%), ≥ 10.0 for adropin (sensitivity 70.0%, specificity 93.3%, PPV 84.0%, NPV 86.2%), ≥ 60.0 for asprosin (sensitivity 83.3%, specificity 83.3%, PPV 71.4%, NPV 90.9%), and ≥ 16.0 for resistin (sensitivity 80.0%, specificity 85.0%, PPV 72.7%, NPV 89.5%) (Table 3).

**Table 2:** Clinicopathological characteristics of malignant breast tumors

Variables (*n* = 30)	
ER positivity, n (%)	28 (93.3)
PR positivity, n (%)	28 (93.3)
HER2 positivity, n (%)	2 (6.7)
AR positivity, n (%)	30 (100.0)
Ki-67 proliferation index, % Median (min-max)	20.0 (1.0–90.0)
p120 catenin expression, n (%)	29 (96.7)
E-cadherin expression, n (%)	25 (83.3)
Perineural invasion, n (%)	12 (40.0)
Lymphovascular invasion, n (%)	13 (43.3)
Associated DCIS	20 (66.7)
Tumor location, n (%)	
Upper outer quadrant Central region Upper inner quadrant Outer region (non-quadrant) Upper region Lower outer quadrant	16 (53.3) 7 (23.3) 3 (10.0) 2 (6.7) 1 (3.3) 1 (3.3)
Tumor size, mm	
Median (min-max)	20.0 (5.0–75.0)
T stage, n (%)	
T1 T2 T3 T4	15 (50.0) 14 (46.7) 0 (0.0) 1 (3.3)
N stage, n (%)	
N0 N1 N2 N3	25 (83.3) 2 (6.7) 0 (0.0) 3 (10.0)

**Table 3:** Diagnostic performance of adiponectin, adropin, asprosin, and resistin for the identification of malignant breast cancer from non-malignant cases

	AUC (95% CI)	*P*	Cut-off	Sensitivity (%)	Specificity (%)	+LHR	PPV (%)	NPV (%)	Max Youden Index
Adiponectin	0.786 (0.693–0.879)	**< 0.001**	≤ 140.0	70.0	76.7	3.0	60.0	83.6	0.467
Adropin	0.893 (0.824–0.963)	**< 0.001**	≥ 10.0	70.0	93.3	10.5	84.0	86.2	0.633
Asprosin	0.906 (0.846–0.966)	**< 0.001**	≥ 60.0	83.3	83.3	5.0	71.4	90.9	0.667
Resistin	0.908 (0.847–0.968)	**< 0.001**	≥ 16.0	80.0	85.0	5.3	72.7	89.5	0.650

*+LHR* Positive Likelihood Ratio, *PPV* Positive Predictive Value, *NPV* Negative Predictive Value

Data in bold is statistically significant

Spearman correlation analysis revealed strong positive correlations among adropin, asprosin, and resistin levels (Table 4). Adropin showed a strong correlation with asprosin (*r* = 0.803, *p* < 0.001) and resistin (*r* = 0.851, *p* < 0.001)). Similarly, asprosin was strongly correlated with resistin (*r* = 0.743, *p* < 0.001)). In contrast, adiponectin demonstrated weak negative correlations with asprosin (*r* = − 0.266, *p* = 0.011) and showed no significant correlations with adropin (*r* = − 0.180, *p* = 0.089) or resistin (*r* = − 0.166, *p* = 0.117). Tumor stage showed moderate negative correlations with adropin (*r* = − 0.438, *p* = 0.015), asprosin (*r* = − 0.408, *p* = 0.025), and resistin (*r* = − 0.420, *p* = 0.021), whereas no significant association was observed with adiponectin. Tumor size was negatively correlated with adiponectin (*r* = − 0.444, *p* = 0.014), adropin (*r* = − 0.419, *p* = 0.021), and resistin (*r* = − 0.379, *p* = 0.039). No significant correlations were found between biomarkers and N stage or Ki-67 index. BMI showed weak positive correlations with adropin (*r* = 0.285, *p* = 0.006), asprosin (*r* = 0.255, *p* = 0.015), and resistin (*r* = 0.209, *p* = 0.048).

**Table 4:** Spearman correlation analysis among serum adiponectin, adropin, asprosin, and resistin levels

Variables	Adiponectin	Adropin	Asprosin	Resistin
Adiponectin	-	−0.180 (0.089)	**−0.266 (0.011)**	−0.166 (0.117)
Adropin	−0.180 (0.089)	-	**0.803 (< 0.001)**	**0.851 (< 0.001)**
Asprosin	**−0.266 (0.011)**	**0.803 (< 0.001)**	-	**0.743 (< 0.001)**
Resistin	−0.166 (0.117)	**0.851 (< 0.001)**	**0.743 (< 0.001)**	-
T stage	-0.312 (0.094)	**-0.438 (0.015)**	**-0.408 (0.025)**	**-0.420 (0.021)**
Tumor size	**-0.444 (0.014)**	**-0.419 (0.021)**	-0.359 (0.051)	**-0.379 (0.039)**
N stage	-0.119 (0.531)	-0.237 (0.207)	-0.342 (0.065)	-0.283 (0.129)
Ki-67 proliferation index	-0.206 (0.275)	0.098 (0.605)	0.113 (0.553)	0.143 (0.451)
BMI	-0.177 (0.095)	**0.285 (0.006)**	**0.255 (0.015)**	**0.209 (0.048)**

Data in bold is statistically significant

Multivariate logistic regression analysis was performed to assess the independent effects of the biomarkers. Since adropin, asprosin, and resistin were highly correlated, separate models were constructed to avoid multicollinearity. BMI showed weak but statistically significant correlations with several biomarkers. Therefore, BMI was included as a covariate in the multivariate models to account for its potential confounding effect. In Model 1, lower adiponectin levels (≤ 140 µg/mL) (OR: 31.3, *p* = 0.001) and higher resistin levels (≥ 16 ng/mL) (OR: 79.9, *p* < 0.001) were independently associated with malignant breast cancer, while BMI was not significant (*p* = 0.794). In Model 2, adiponectin (OR: 30.8, *p* = 0.001) and asprosin (OR: 85.6, *p* < 0.001) both showed strong independent associations with malignancy. Similarly, in Model 3, adiponectin (OR: 14.6, *p* = 0.001) and adropin (OR: 53.9, *p* < 0.001) remained significant predictors (Table 5).

**Table 5:** Multivariate logistic regression models for the prediction of malignant breast cancer

Model	Variable	OR	95% CI	*p*
Model 1	Adiponectin (≤ 140 vs. >140)	31.3	3.8-256.2	0.001
	Resistin (≥ 16 vs. <16)	79.9	9.3-682.3	< 0.001
	BMI	0.97	0.84–1.15	0.794
Model 2	Adiponectin (≤ 140 vs. >140)	30.8	3.7-253.3	0.001
	Asprosin (≥ 60 vs. <60)	85.6	9.9–740.0	< 0.001
	BMI	0.98	0.83–1.15	0.805
Model 3	Adiponectin (≤ 140 vs. >140)	14.6	3.0-72.4	0.001
	Adropin (≥ 10 vs. <10)	53.9	9.5-304.9	< 0.001
	BMI	1.02	0.87–1.19	0.778

*OR *Odds ratio,* CI *Confidence interval,* BMI *Body mass index

models were constructed separately to avoid multicollinearity due to high correlations among adropin, asprosin, and resistin

## Discussion

Significant advancements have been made in the diagnosis and treatment of breast cancer. Although radiological screening methods used for early diagnosis have established sensitivity and specificity, the addition of biochemical markers may help improve diagnostic accuracy. In particular, the early detection of ductal carcinoma in situ (DCIS) and early-stage breast cancer remains challenging, as imaging techniques may still present diagnostic challenges [17]. Imaging modalities remain the cornerstone of breast cancer detection and staging. Breast MRI has demonstrated very high sensitivity, reaching up to 90–100% in certain clinical settings, and is recommended in selected patients where additional information may influence treatment decisions [18, 19]. However, despite its high sensitivity, MRI is not universally applied and is typically used on a case-by-case basis due to considerations such as cost, availability, and potential false-positive findings. In this context, circulating biomarkers may provide complementary information by reflecting systemic metabolic and inflammatory alterations that are not captured by imaging alone. Therefore, integrating imaging findings with circulating adipokines may represent a promising multimodal approach to improve diagnostic accuracy and clinical decision-making in breast cancer. Thus, these biomarkers are not suitable for standalone screening but may serve as adjunctive diagnostic tools to complement imaging modalities. In light of clinical evidence demonstrating the detrimental impact of obesity on breast cancer development and outcomes [20], the incorporation of adipose tissue–derived biomarkers into current early diagnostic frameworks may offer added clinical utility.

In this study, we investigated the diagnostic value of serum adiponectin, adropin, asprosin, and resistin levels in patients with malignant breast cancer compared with benign breast disease and healthy controls. Our results showed that adropin, asprosin, and resistin levels were significantly elevated in malignant cases and demonstrated good to excellent discriminative performance for identifying malignancy. In contrast, adiponectin levels were significantly lower in malignant breast cancer compared with non-malignant groups but exhibited a more moderate diagnostic performance. Correlation analyses further indicated that while adropin, asprosin, and resistin were associated with tumor stage, tumor size, and body mass index, adiponectin showed weaker and more selective associations with tumor-related parameters. Collectively, these findings suggest that adiponectin may primarily reflect metabolic alterations associated with malignancy, whereas adropin, asprosin, and resistin appear to better capture tumor-related biological and inflammatory processes, supporting their potential complementary role in the diagnostic evaluation of breast cancer.

Prior studies have consistently demonstrated that lower circulating adiponectin levels are associated with an increased risk of breast cancer, especially in large case–control and cohort series, supporting an inverse relationship between adiponectin concentration and breast cancer occurrence [21]. For example, Miyoshi and colleagues reported that women in the lowest tertile of serum adiponectin had a significantly higher risk of breast cancer compared with those in the highest tertile, and lower adiponectin was linked to more aggressive tumor features such as larger size and higher grade [22]. Meta-analysis evidence further suggests that serum adiponectin values are inversely associated with breast cancer risk, with pooled analyses showing lower adiponectin levels in cases than in controls across multiple populations [6]. Similarly, studies conducted on Asian women have reported lower serum adiponectin levels in breast cancer patients [23]. Some case–control studies have also explored adiponectin’s prognostic relevance, indicating that higher adiponectin levels at diagnosis may be associated with better disease-free survival in subsets of women, particularly those with normal body weight [24]. In contrast to these broader epidemiological findings, our study observed that adiponectin exhibited a more moderate diagnostic performance compared with adropin, asprosin, and resistin in distinguishing malignant from non-malignant cases, and its associations with clinicopathological variables were comparatively weaker. This difference may reflect the fact that adiponectin is influenced by metabolic and adiposity-related factors in addition to tumor biology, and that its relationship with malignancy risk and outcome may vary by menopausal status, BMI, and other confounders as highlighted in the literature [25].

Emerging evidence suggests that adropin may play a role in cancer biology through its involvement in metabolic and inflammatory pathways. In endometrial cancer, serum adropin levels have been reported to be significantly lower in malignant cases compared with controls, suggesting a potential role in disease pathophysiology [11]. Given its role in glucose and lipid metabolism, alterations in adropin levels may reflect tumor-associated metabolic changes rather than representing a tumor-specific marker. In our study, adropin levels showed a weak but statistically significant positive correlation with BMI, supporting the influence of metabolic status on circulating levels. Although clinical studies directly evaluating adropin in breast cancer remain limited, experimental data suggest that adropin may affect cancer cell viability and apoptosis, indicating a potential mechanistic link with tumor biology [26]. Similar to adropin, research on asprosin in breast cancer remains limited but growing. Initial studies have found higher circulating asprosin levels in breast cancer patients compared with non-malignant groups, and associations between asprosin and tumor grade have been reported, suggesting that asprosin might serve as a marker of tumor presence and possibly tumor burden [27]. Another study investigating the role of asprosin in breast cancer diagnosis has also been conducted. These studies reported that serum asprosin levels were elevated in breast cancer patients but emphasized the need for further research to confirm its utility as a biomarker [28]. In our study, asprosin exhibited excellent discriminative accuracy and favorable diagnostic metrics, further supporting its potential role as a complementary biomarker in breast cancer evaluation. In contrast to both adropin and asprosin, resistin has a more established literature base in breast cancer, with multiple clinical studies showing elevated resistin levels in malignant cases and associations with markers of inflammation, advanced tumor stage, and unfavorable outcomes [29, 30]. A systematic review and meta-analysis on breast cancer demonstrated that resistin levels were significantly higher in breast cancer patients compared to healthy individuals. This study also identified BMI as a crucial moderator in the relationship between resistin and breast cancer, suggesting that resistin may contribute to breast cancer risk through obesity-related mechanisms [31]. Our findings that resistin levels were significantly higher in malignant patients and that resistin demonstrated robust ROC performance are consistent with this literature, reinforcing its role as both a marker of systemic inflammation and a possible indicator of tumor aggressiveness. Taken together, while adiponectin appears to be primarily influenced by broader metabolic status and may have a limited direct diagnostic value in breast cancer, adropin, asprosin, and resistin consistently show stronger associations with malignancy. These biomarkers may capture different aspects of tumor biology, including metabolic dysregulation and inflammatory responses, and thus hold potential as complementary diagnostic tools to enhance risk stratification beyond traditional markers.

A notable strength of the present study is the simultaneous evaluation of adiponectin, adropin, asprosin, and resistin within the same breast cancer cohort. While previous studies have predominantly focused on individual adipokines or limited biomarker panels, evidence integrating these four metabolically and inflammatory relevant biomarkers in a single clinical setting is scarce [26, 27, 32]. By assessing their diagnostic performance, inter-marker correlations, and associations with clinicopathological parameters concurrently, our study provides a more comprehensive overview of their relative and complementary roles in breast cancer. This integrated approach allows for a clearer interpretation of shared and distinct biological signals, particularly in the context of metabolic and inflammatory alterations accompanying malignancy and may help inform future multi-marker strategies for breast cancer evaluation.

Several analytical considerations should be acknowledged when interpreting the present findings. The relatively small sample size of this study may limit the generalizability of the findings. The high ORs and wide confidence intervals observed in the regression analyses may be explained by the relatively small sample size and the dichotomization of continuous variables using ROC-derived cut-off values. These methodological factors may have contributed to an overestimation of effect sizes, and thus, the findings should be interpreted with caution. Strong correlations were observed among adropin, asprosin, and resistin. When predictors are highly correlated, multivariable analyses may produce unstable estimates and limit the ability to clearly distinguish independent effects. To address this, separate multivariable models were constructed in combination with adiponectin, avoiding the inclusion of highly correlated variables in the same model. Although all biomarkers showed good diagnostic performance, their intercorrelations suggest overlapping biological pathways. Therefore, their independent effects should be interpreted with caution.

These biomarkers are not specific to breast cancer and may also be altered in other malignancies, inflammatory conditions, and metabolic disorders. Therefore, their pathognomonic value remains limited. Future studies with larger cohorts and alternative modeling approaches, such as dimension reduction or composite biomarker scores, are needed to clarify their relative and independent contributions.

## Data Availability

All data generated or analyzed during this study are included in this article. Further enquiries can be directed to the corresponding author.

## Abbreviations

MRI: Magnetic resonance imaging
SD: Standard deviation
ROC: Receiver operating characteristic
AUC: Area under the ROC curve
LHR: Likelihood ratio
PPV: Positive predictive value
NPV: Negative predictive value
BMI: Body Mass Index
ER: Estrogen receptor
PR: Progesteron Receptor
HER2: Human Epidermal Growth Factor Receptor 2
AR: Androgen Receptor
PNI: Perineural invasion
LVI: Lymphovascular invasion
DCIS: Ductal carcinoma in situ
